# Oral administration of fructose exacerbates liver fibrosis and hepatocarcinogenesis via increased intestinal permeability in a rat steatohepatitis model

**DOI:** 10.18632/oncotarget.25587

**Published:** 2018-06-19

**Authors:** Kenichiro Seki, Mitsuteru Kitade, Norihisa Nishimura, Kosuke Kaji, Kiyoshi Asada, Tadashi Namisaki, Kei Moriya, Hideto Kawaratani, Yasushi Okura, Hiroaki Takaya, Yasuhiko Sawada, Shinya Sato, Keisuke Nakanishi, Hitoshi Yoshiji

**Affiliations:** ^1^ Third Department of Internal Medicine, Nara Medical University, Nara, Japan

**Keywords:** non-alcoholic steatohepatitis (NASH), fructose, hepatocarcinogenesis, intestinal permeability, endotoxin

## Abstract

Recent reports have revealed the impact of a western diet containing large amounts of fructose on the pathogenesis of non-alcoholic steatohepatitis (NASH). Fructose exacerbates hepatic inflammation in NASH by inducing increasing intestinal permeability. However, it is not clear whether fructose contributes to the progression of liver fibrosis and hepatocarcinogenesis in NASH. The aim of this study was to investigate the effect of fructose intake on NASH in a rat model. A choline-deficient/L-amino acid diet was fed to F344 rats to induce NASH. Fructose was administrated to one group in the drinking water. The development of liver fibrosis and hepatocarcinogenesis were evaluated histologically. Oral fructose administration exacerbated liver fibrosis and increased the number of preneoplastic lesions positive for glutathione S-transferase placental form. Fructose-treated rats had significantly higher expression of hepatic genes related to toll-like receptor-signaling, suggesting that fructose consumption increased signaling in this pathway, leading to the progression of NASH. We confirmed that intestinal permeability was significantly higher in fructose-treated rats, as evidenced by a loss of intestinal tight junction proteins. Fructose exacerbated both liver fibrosis and hepatocarcinogenesis by increasing intestinal permeability. This observation strongly supports the role of endotoxin in the progression of NASH.

## INTRODUCTION

Nonalcoholic fatty liver disease (NAFLD) is increasingly common, its prevalence rising in parallel with that of the metabolic syndrome under the influence of a western diet. Nonalcoholic fatty liver is a condition without inflammation or fibrosis but which may progress to nonalcoholic steatohepatitis (NASH) and liver cirrhosis [1]. The multiple parallel hits hypothesis suggests that various factors, including the metabolic syndrome with visceral fat accumulation or insulin resistance, oxidative stress, or endotoxins such as lipopolysaccharide (LPS), act synchronously on the liver to induce NASH [2]. However, detailed mechanisms of these posited multiple parallel hits have not yet been fully elucidated.

Recent research has also evaluated the impact of a western diet on NASH pathogenesis. This diet typically contains a large amount of fructose, a monosaccharide widely used as a sweetener [3]. A strong relationship between the development of NASH and high fructose consumption has been reported [4–7]. Fructose intake is associated with insulin resistance and fat accumulation in the liver [8, 9]. Mice chronically fed with a fructose-rich diet were found to have increased endotoxin levels in the portal blood and activation of the toll-like receptor (TLR) 4-nuclear factor-kappa B (NF-κB) signal cascade mainly in Kupffer cells, playing a central roles on hepatic innate immunity [10, 11]. It has also been reported that concomitant treatment with antibiotics almost blocked the effect of fructose on the mouse liver [6] as well as on rat metabolic syndrome, inflammation and oxidative stress [12]. It has further been shown that chronic intake of fructose is associated with a decrease in tight junction proteins (TJPs) in the small intestine. In addition, fructose is reported to worsen inflammation in clinical NASH [13, 14]. Based on these findings, it is now clear that high fructose intake exacerbates hepatic inflammation in NASH by inducing intestinal permeability and increased portal blood LPS concentrations. However, the mechanism by which fructose contributes to the progression of liver fibrosis and hepatocarcinogenesis in NASH has yet to be clarified.

In the present study, we employed a rat model of NASH and fed the rats with fructose to observe its effect on the progression of liver fibrosis in NASH.

## RESULTS

### General findings

The findings in each experimental group at the time of sacrifice are shown in Table 1. Administration of choline-deficient/L-amino acid (CDAA) diet significantly increased NAFLD activity score (NAS). Mean body weights in both the CDAA and CDAA + fructose groups were significantly lower than those in the choline-supplemented/L-amino acid (CSAA) group. The CDAA + fructose group weighed significantly less than the CDAA group. In contrast, the mean liver weight of the CDAA group was significantly higher than that of the other two groups. The liver-to-body weight ratio in both CDAA-fed groups was significantly higher than that of the CSAA group.

**Table 1:** Characteristics of a nonalcoholic steatohepatitis model in rats fed different diets

	CSAA	CDAA	CDAA + fructose
Body weight (g)	267.8 ± 12.0	224.5 ± 11.4^a^	189.2 ± 9.7^a,b^
Liver weight (g)	7.9 ± 0.5	12.0 ± 0.5^a^	8.6 ± 0.9^b^
Liver-to-body weight ratio (%)	2.9 ± 0.1	5.3 ± 0.2^a^	4.5 ± 0.4^a,b^
Total caloric intake over 10 weeks (kcal)	5017	3981	3677
Caloric intake from fructose (%)	18.3	18.8	30.0
AST (IU/l)	96.8 ± 12.5	385.7 ± 30.1^a^	475.2 ± 36.0^a,b^
ALT (IU/l)	58 ± 6.4	264.2 ± 40.0^a^	182.3 ± 23.8^a,b^
Albumin (g/dl)	4.7 ± 0.11	4.1 ± 0.11^a^	3.5 ± 0.15^a,b^
Total bilirubin (ng/dl)	0.02 ± 0.01	0.13 ± 0.02^a^	0.38 ± 0.09^a,b^
Glucose (mg/dl)	154.5 ± 29.3	114.7 ± 13.3^a^	98.7 ± 14.3^a^
Insulin (ng/ml)	2.25 ± 0.48	0.53 ± 0.25^a^	0.15 ± 0.05^a,b^
Triglyceride (mg/dl)	81.5 ± 28.2	8.0 ± 1.0^a^	9.0 ± 1.1^a^
NAFLD activity score	0	5.8 ± 0.7^a^	6.2 ± 0.7^a^

Abbreviations: AST, aspartate aminotransferase; ALT, alanine aminotransferase; CDAA, choline-deficient/L-amino acid;

CSAA, choline-supplemented/L-amino acid

Data are presented as the mean ± standard deviation.

^a^P < 0.05 compared with CSAA group

^b^P < 0.05 compared with CDAA group

Total calorie and water intake is each diet was lower in the CDAA-fed groups compared with the CSAA group. Administration of fructose in the drinking water increased the percentage of calories with fructose as a source.

The CDAA + fructose group had higher aspartate aminotransferase but lower alanine aminotransferase levels compared with the CDAA group. The CDAA + fructose group had a slightly lower albumin and a slightly higher total bilirubin compared with the CDAA group, suggesting a greater degree of liver injury in the CDAA + fructose group than in the CDAA group. There were no statistically significant differences in blood glucose, insulin, triglyceride levels and NAFLD activity score between CDAA and CDAA + fructose group. We also have examined rats fed with CSAA diet with oral administration of fructose, but we did not find any significant differences both in serologically and pathologically (data not shown).

### Effect of fructose on liver fibrosis

**Figure 1:**
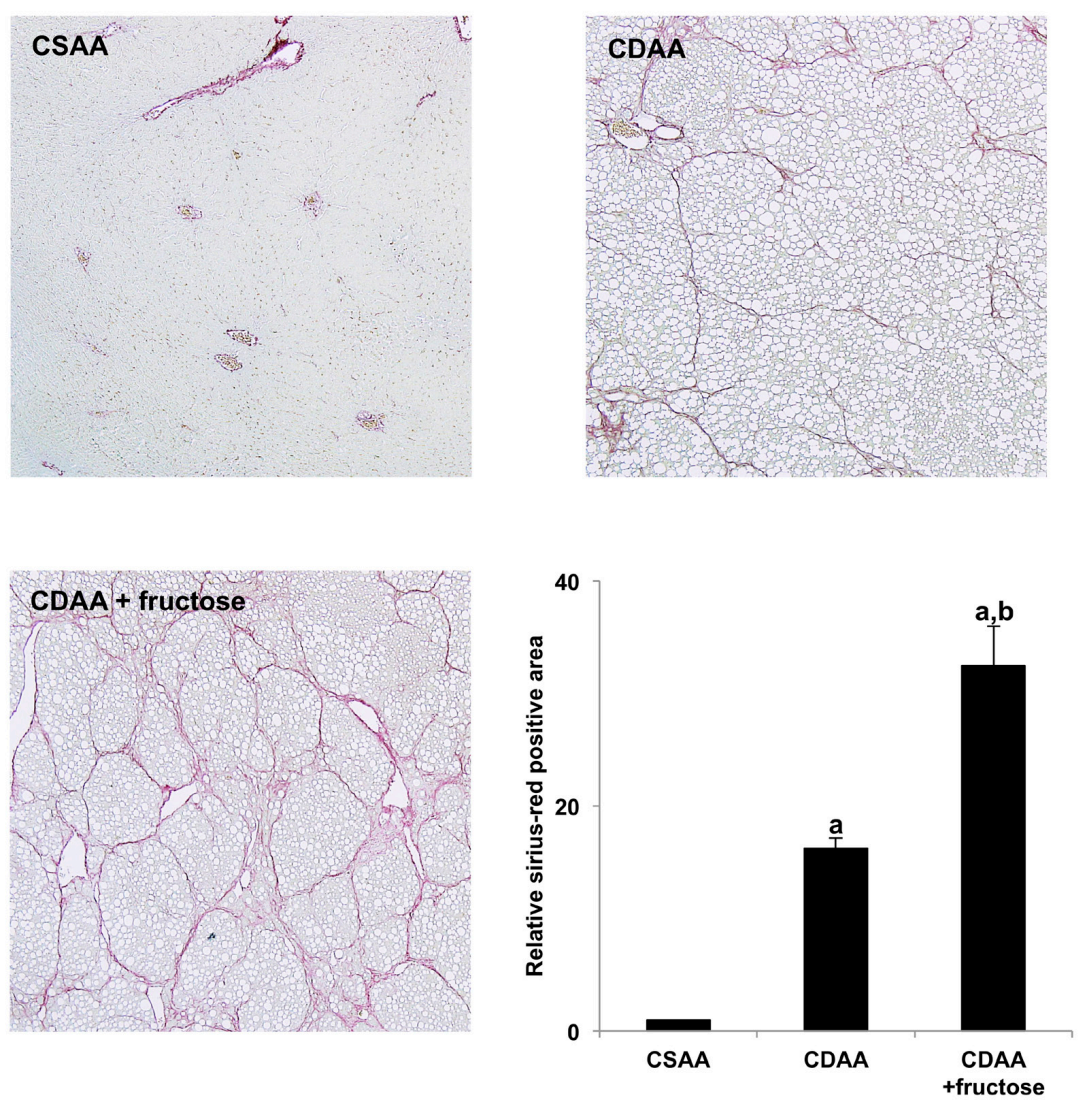
Fructose exacerbates CDAA-induced liver fibrosis Collagen deposition was evaluated by sirius-red staining. Original magnification x40. The CSAA group has no fibrosis, but extensive fibrosis is present in both CDAA-fed groups and is greatest in the CDAA + fructose group. Semi-quantitative analysis confirms the histologic findings. Data are presented as the mean ± standard deviation. ^a^P < 0.05 compared with CSAA group; ^b^P < 0.05 compared with CDAA group. CDAA, choline-deficient/L-amino acid; CSAA, choline-supplemented/L-amino acid.

As shown in Figure 1, sirius-red staining of liver tissue showed no fibrosis in the CSAA group. In sharp contrast, marked fibrosis was observed in both CDAA-fed groups, with the CDAA + fructose group on semi-quantitative analysis having significantly more fibrosis than the CDAA group.

**Figure 2:**
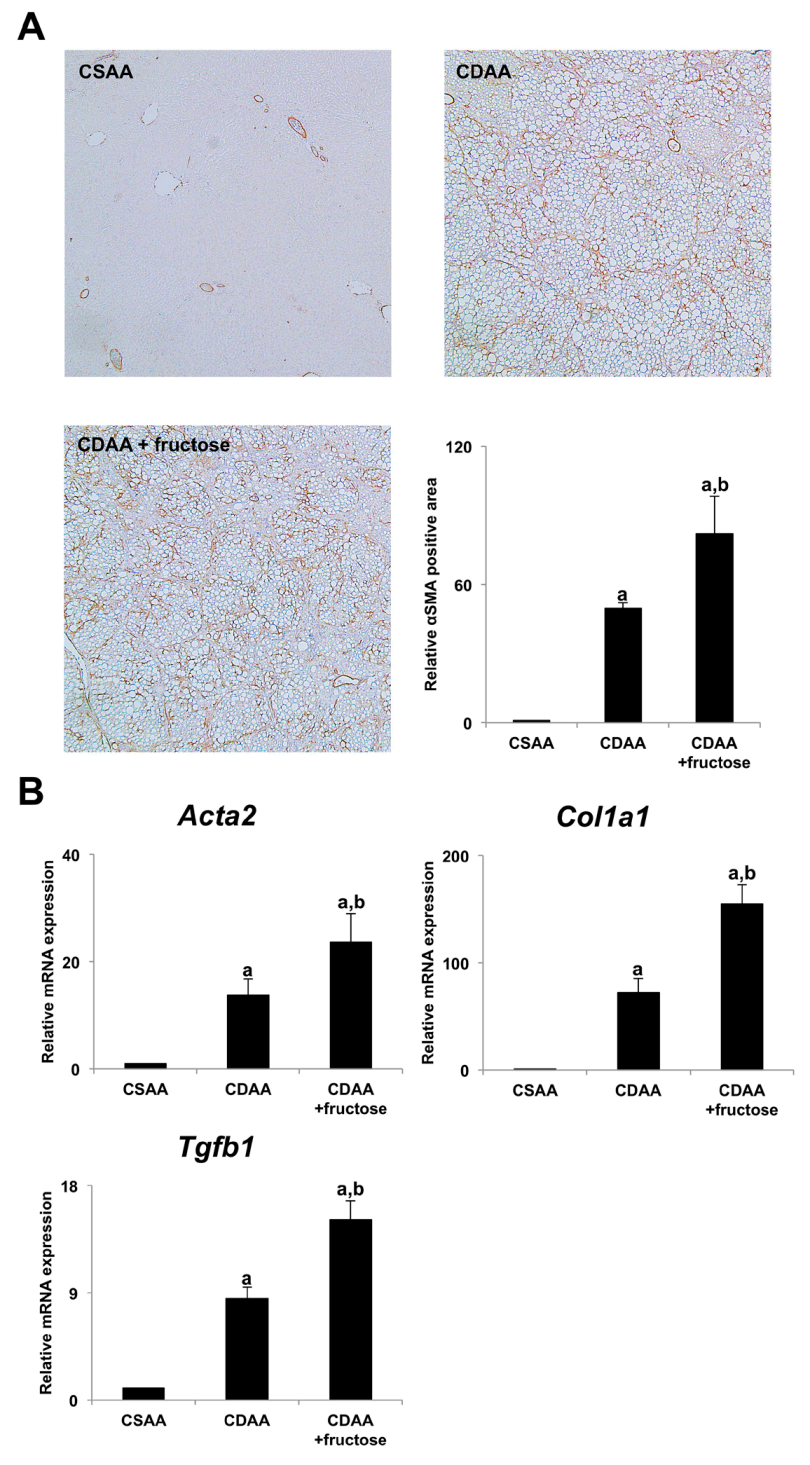
Fructose accelerated hepatic stellate cells-induced liver fibrogenesis **(A)** No αSMA immunopositive cells are observed in the CSAA group. The area occupied by αSMA immunopositive cells is high in both CDAA-fed groups, with the largest area seen in the CDAA + fructose group. Original magnification x40. Semi-quantitative analysis confirms significant differences between the groups. **(B)** The expression of αSMA mRNA (*Acta2*) in the liver is significantly higher in both CDAA-fed groups when compared with the CSAA group, with the highest expression seen in the CDAA + fructose group. Findings for expression of transforming growth factor beta 1 (*Tgfb1*) and collagen-Iα (*Col1a1*) mRNA in the liver are similar. Data are presented as the mean ± standard deviation. ^a^P < 0.05 compared with CSAA group; ^b^P < 0.05 compared with CDAA group. CDAA, choline-deficient/L-amino acid; CSAA, choline-supplemented/L-amino acid; αSMA, alpha smooth muscle actin.

Immunohistochemical analysis of α-smooth muscle actin (αSMA) staining was performed to evaluate the activation of hepatic stellate cells (HSCs) which plays a central role in liver fibrogenesis. Both CDAA-fed groups again had significantly greater αSMA-positive areas, with the greatest area in the CDAA + fructose group (Figure 2A). Additionally, expression of pro-fibrotic genes, including *Acta2*, *Col1a1*, and *Tgfb1* was significantly higher the CDAA and CDAA + fructose groups in parallel with the differences in liver fibrosis and numbers of activated HSCs (Figure 2B). These observations indicate that addition of fructose augmented liver fibrosis to a greater degree than did the CDAA diet alone.

### Effect of fructose on liver carcinogenesis

**Figure 3:**
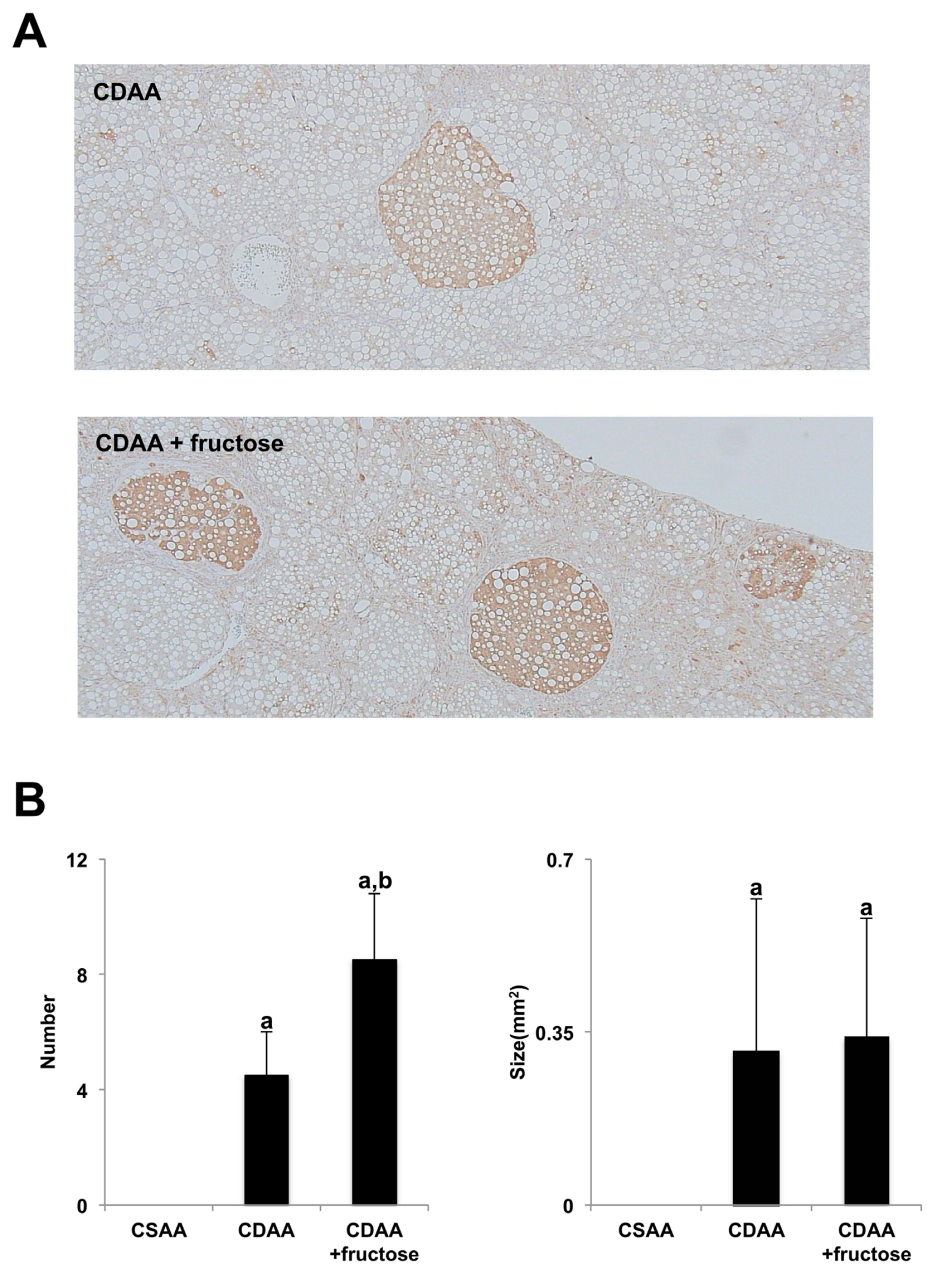
Fructose augmented CDAA-induced hepatocarcinogenesis **(A)** GST-P-positive foci are seen in both CDAA-fed groups. Original magnification x40. **(B)** Although the mean number of GST-P-positive foci is highest in the CDAA + fructose group, the mean size does not differ between the two CDAA-fed groups. Data are presented as the mean ± standard deviation. ^a^P < 0.05 compared with the CSAA group; ^b^P < 0.05 compared with the CDAA group. CDAA, choline-deficient/L-amino acid; CSAA, choline-supplemented/L-amino acid; GST-P, glutathione S-transferase placental form.

Liver carcinogenesis was evaluated by measuring the number and size of glutathione S-transferase placental form (GST-P)-positive lesions. As shown in Figure 3, rats fed the CDAA diet had a greater number GST-P-positive foci than those fed the CSAA diet, and the addition of fructose resulted in a significantly higher number of these preneoplastic foci. This suggests that a fructose-rich diet may induce hepatocarcinogenesis in NASH.

### Fructose did not affect fat accumulation or lipid peroxidation in the liver

**Figure 4:**
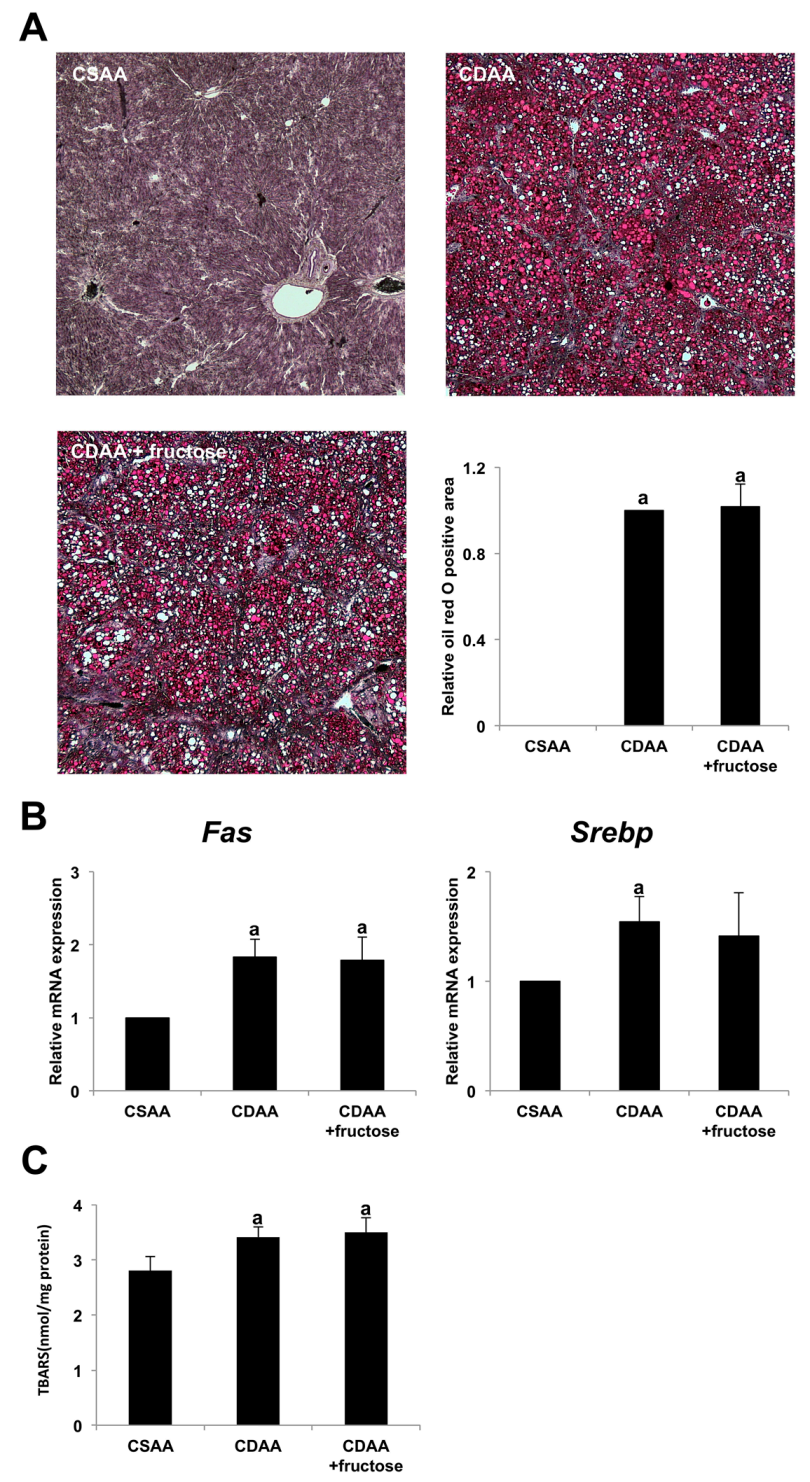
The effect of fructose administration on hepatic steatosis in the CDAA-induced steatohepatitis **(A)** Fat droplet deposition indicated by oil red O staining. Original magnification x40. None is seen in the CSAA group, but it is conspicuous in both CDAA-fed groups Semi-quantitative analysis indicates no significant difference between the two CDAA-fed groups. **(B)** The expression of *Fas* mRNA and *Srebp* mRNA in the liver does not differ significantly between the two CDAA-fed groups. (C) Oxidative stress of liver tissue as evaluated by TBARS is significantly higher in the two CDAA-fed groups than the CSAA group. However, TBARS expression does not differ significantly between the two CDAA-fed groups. Data are presented as the mean ± standard deviation. ^a^P < 0.05 compared with the CSAA group. CDAA, choline-deficient/L-amino acid; CSAA, choline-supplemented/L-amino acid; TBARS, thiobarbituric acid reactive substances.

We evaluated whether the degree of hepatic steatosis was associated with the higher degree of liver fibrosis in the CDAA + fructose group. Although oil red O staining of liver tissue showed marked steatosis in both CDAA-fed groups, there was no significant difference between these two groups (Figure 4A). In parallel with the histologic observation, administration of fructose did not affect hepatic expression of the lipogenic genes *Srebp* and *Fas* (Figure 4B).

Lipid peroxidation induced by CDAA with or without fructose was measured by assessing thiobarbituric acid reactive substances (TBARS). Similar to the appearance of hepatic steatosis, the CDAA diet induced hepatic TBARS, but addition of fructose was not associated with a significant difference in TBARS levels (Figure 4C). Taken together, these observations suggest that the higher degree of liver fibrogenesis associated with fructose administration in CDAA-induced steatohepatitis was not caused by either hepatic steatosis or an increase in reactive oxygen species (ROS).

### Fructose administration increased intestinal permeability by decreasing TJP expression

**Figure 5:**
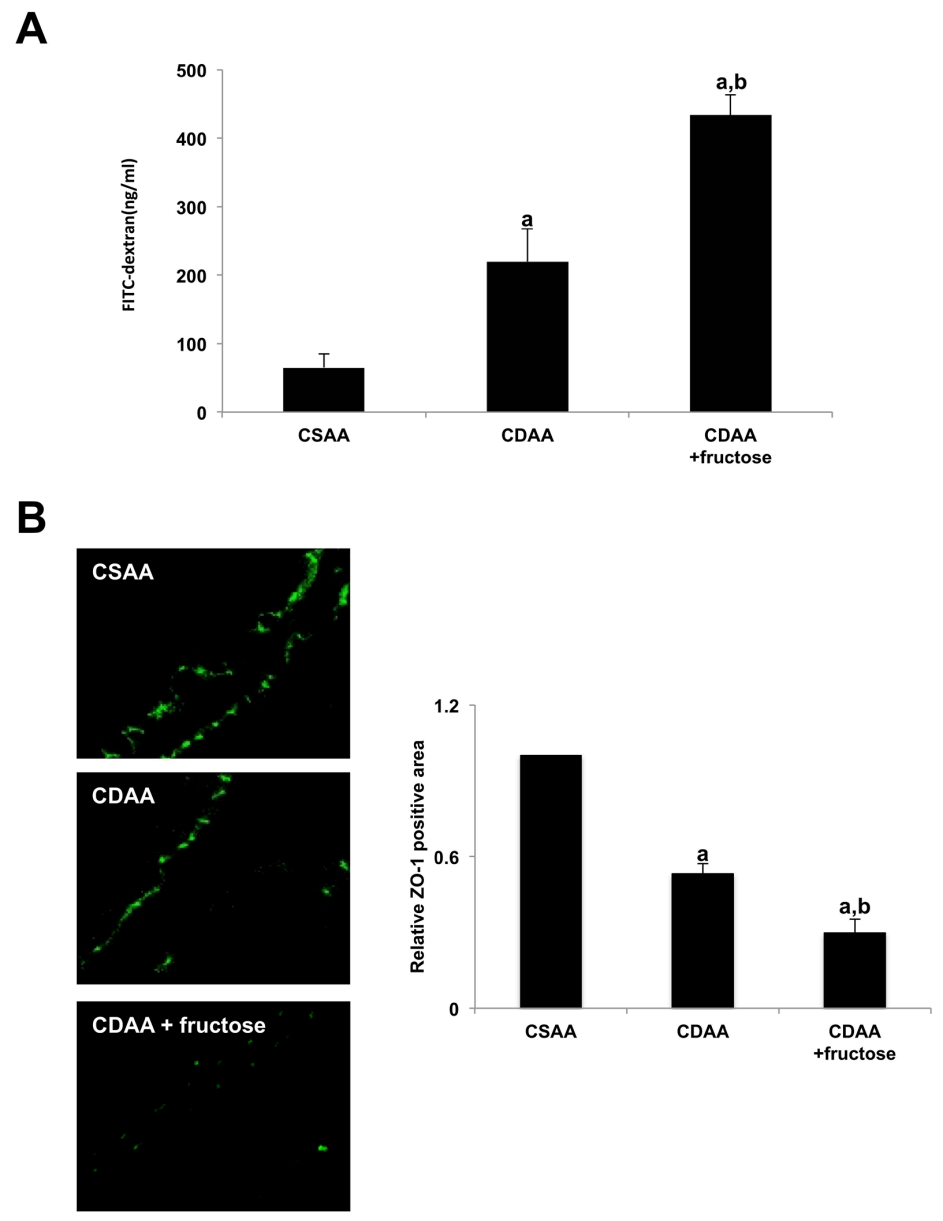
Fructose exacerbates intestinal permeability in rats **(A)** The fluorescein level in the portal vein after FITC-dextran administration is significantly higher in both CDAA-fed groups compared with the CSAA group and is significantly higher in the CDAA + fructose group than the CDAA group. **(B)** Fructose attenuates TJPs in the small intestine. Immunohistochemical staining demonstrates adequate expression of ZO-1 in intestinal sections of the CSAA group compared with both CDAA-fed groups. Fructose further attenuates TJP expression as demonstrated by semi-quantitative analysis. Original magnification x400. Data are presented as the mean ± standard deviation. ^a^P < 0.05 compared with the CSAA group; ^b^P < 0.05 compared with the CDAA group. CDAA, choline-deficient/L-amino acid; CSAA, choline-supplemented/L-amino acid; FITC, fluorescein isothiocyanate; TJP, tight junction protein; ZO-1, zonula occludens-1.

We next focused on the gut-liver axis. Intestinal permeability was examined by measuring fluorescence levels in portal vein blood following oral gavage with fluorescein isothiocyanate (FITC)-dextran. Both CDAA-fed groups had higher FITC positivity than did the CSAA group, indicating increased intestinal permeability. The CDAA + fructose group had a significantly higher level of portal FITC intensity, suggesting that fructose independently exacerbates intestinal permeability (Figure 5A).

Next, to assess intestinal permeability, we evaluated expression of zonula occludens-1 (ZO-1), one of the major tight junction proteins in the intestine by immunofluorescence (Figure 5B). The intestinal expression of ZO-1 was clearly observed on the apical side of the intestinal mucosa in the CSAA group. Intestinal ZO-1 positivity was lower in the CDAA group and lowest in the CDAA + fructose group, with the differences between each group being statistically significant. These observations suggest that oral fructose was responsible for increased intestinal permeability by reducing the expression of TJPs.

### Effect of fructose on LPS-TLR4 signaling

**Figure 6:**
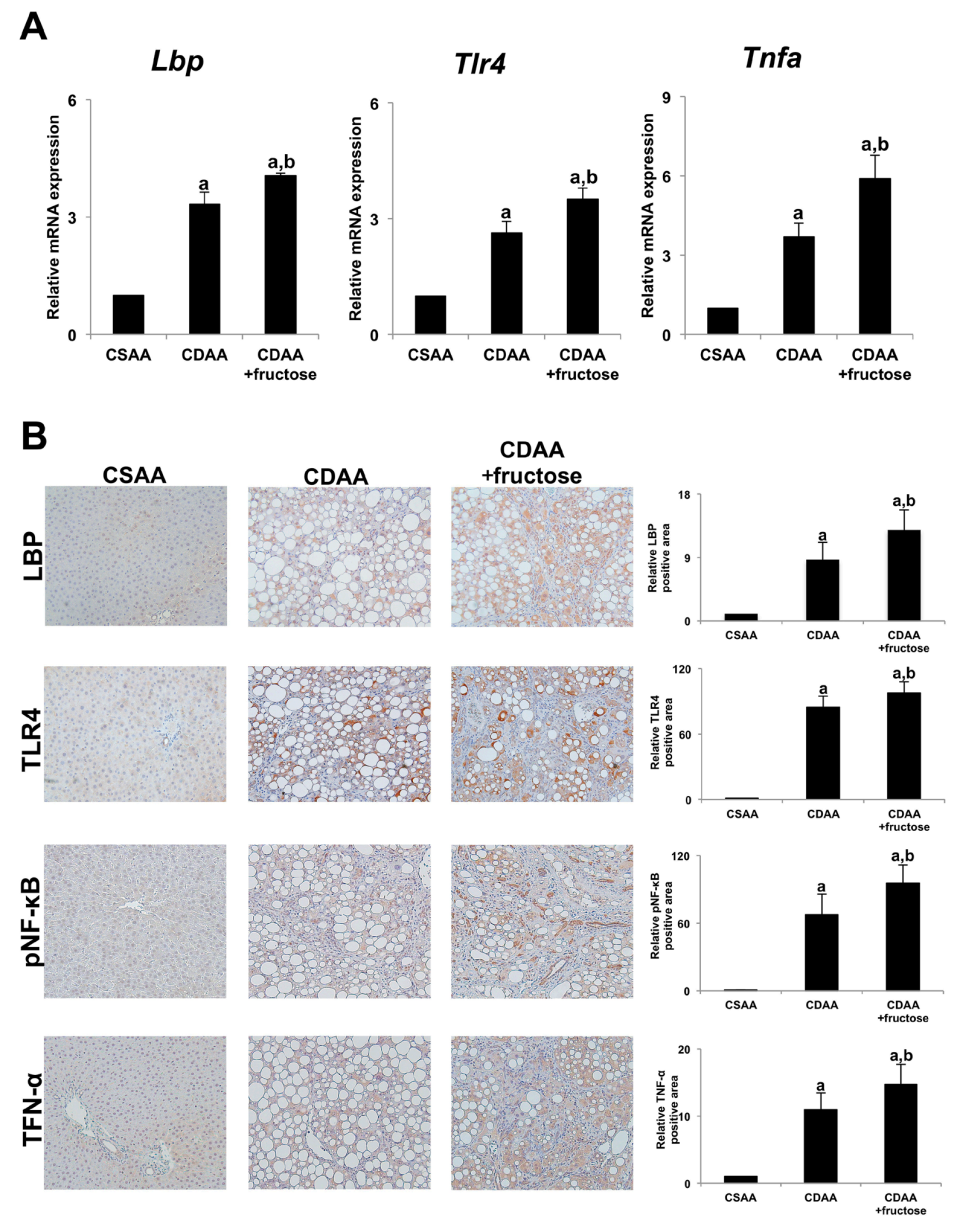
Fructose enhances LPS-TLR4 signaling in rat liver **(A)** The expression of *Lbp* mRNA, *Tlr4* mRNA, and *Tnfa* mRNA are significantly greater in both CDAA-fed groups compared with the CSAA group, and that in the CDAA + fructose group is significantly higher than in the CDAA group. **(B)** Immunohistochemistry of LBP, TLR4, pNF-κB and TNF-α. Original magnification x100. The hepatic expressions of each molecule are significantly higher in both CDAA-fed groups compared with the CSAA group, and that in the CDAA + fructose group is significantly higher than in the CDAA group. Data are presented as the mean ± standard deviation. ^a^P < 0.05 compared with the CSAA group; ^b^P < 0.05 compared with the CDAA group. CDAA, choline-deficient/L-amino acid; CSAA, choline-supplemented/L-amino acid; LBP, LPS-binding protein; LPS, lipopolysaccharide; TLR4, toll-like receptor 4; TNF-α, tumor necrosis factor alpha; pNF-κB, phospho-nuclear factor-kappa B.

Based upon the observation that oral administration of fructose increased intestinal permeability, we examined whether a fructose-induced “leaky gut” may increase portal blood levels of endotoxin, particularly LPS, leading to exacerbation of NASH. Since it is difficult to measure LPS in the portal blood, we examined hepatic expression of LPS-binding protein (LBP), which is reported to correlate with serum LPS levels [15]. Hepatic expression of *Lbp* as well as the related downstream signaling genes *Tlr4* and *Tnfa* was higher in the CDAA group and even more so in the CDAA + fructose group (Figure 6A). We also evaluated co-localization of LPS-TLR4 signal-related molecules i.e. LBP, TLR4, phospho-nuclear factor-kappa B (pNF-κB) and Tumor necrosis factor alpha (TNF-α) by immunohistochemistry (Figure 6B). The expressions of each molecules in the liver were significantly elevated in CDAA-fed rats when compared with the CSAA group, and oral fructose administration further increased expression of these molecules in the liver in parallel with progression of liver pathogenesis i.e. liver fibrosis development or number of GST-P positive hepatic preneoplastic lesions (Figure 6B). These findings suggest that fructose enhanced the intrahepatic TLR4-LPS signaling cascade by way of increased portal blood LPS levels, thus indicating a mechanism by which fructose consumption contributes to the progression of NASH.

### Leaky gut-derived hepatic LPS-TLR4-NF-κB signals interact with both Kupffer cells and activated HSC (Ac-HSC)

**Figure 7:**
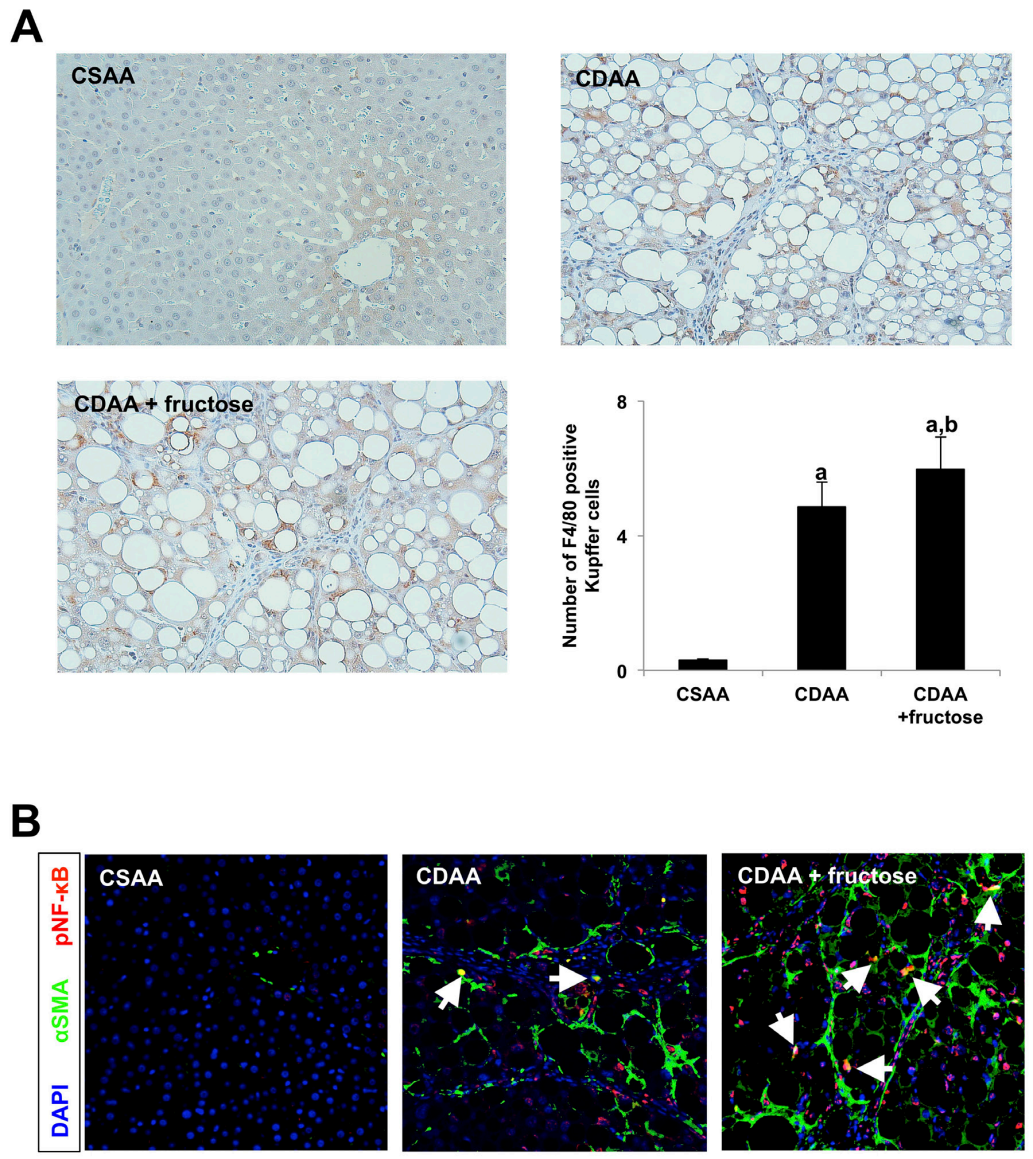
Leaky gut-derived hepatic LPS-TLR4-NF-κB signals interact with both Kupffer cells and Ac-HSC **(A)** Immunohistochemistry of F4/80. Original magnification x100. F4/80 positive Kupffer cells are significantly increased in CDAA-fed rats when compared with the CSAA group, and oral fructose administration further increased the number of Kupffer cells in the liver. Graph shows the number of F4/80 positive Kupffer cells per field. **(B)** Confocal images of livers labeled by αSMA (green), nuclear (blue) and pNF-κB (red). Original magnification x100. Arrows indicate both pNF-κB and αSMA positive Ac-HSC. The number of pNF-κB positive Ac-HSC is remarkably increased when oral fructose is administrated with CDAA diet. Data are presented as the mean ± standard deviation. ^a^P < 0.05 compared with the CSAA group; ^b^P < 0.05 compared with the CDAA group. CDAA, choline-deficient/L-amino acid; CSAA, choline-supplemented/L-amino acid; αSMA, alpha smooth muscle actin; pNF-κB, phospho-nuclear factor-kappa B.

Finally, we examined whether leaky gut-derived hepatic LPS-TLR4-NF-κB signal interact with Ac-HSC as well as Kupffer cells. Our immunohistochemistry showed that F4/80 positive Kupffer cells are significantly increased in CDAA-fed rats when compared with the CSAA group, and oral fructose administration further increased the number of Kupffer cells in the liver (Figure 7A). We then performed immunofluoresence of both αSMA (green) and pNF-κB (red) to confirm whether LPS-TLR4-NF-κB signal is involved in Ac-HSC as well as Kupffer cells. Confocal images showed a significant increase of pNF-κB positive non-parenchymal cells along with liver inflammation or fibrotic septa reflecting the increase of Kupffer cells in both CDAA-fed group and CDAA + fructose group (Figure 7B). Among them, pNF-κB and αSMA double positive cells indicating pNF-κB positive Ac-HSC were observed in CDAA-fed group. The number of pNF-κB positive Ac-HSC was remarkably increased when oral fructose administration with CDAA diet was performed (Figure 7B). These results suggest that LPS affects not only Kupffer cells but also HSCs which resulted in advanced liver fibrosis development by oral fructose administration.

## DISCUSSION

Recent research has revealed the impact of a western diet, particularly high fructose consumption, on augmentation of inflammation in NASH related to various factors, such as insulin resistance, hepatic fat accumulation, or increases intestinal permeability [3]. However, it has not been proven whether fructose intake promotes progression of NASH by inducing liver fibrosis or hepatocarcinogenesis. Here we have demonstrated for the first time that fructose exacerbates liver fibrosis, and potentially hepatocarcinogenesis as well, in a rodent NASH model.

In the current experiment, fructose administration increased intestinal permeability with a loss of TJPs of intestinal mucosal cells, as reported previously [16]. TJPs, including ZO-1, are intercellular adhesion factors which influence the transit of various substances from the intestinal lumen to the blood [17, 18]. When intestinal permeability is increased, endotoxin flows into the liver from the portal vein and stimulates the endotoxin receptor on Kupffer cells, TLR4, which plays a central role in liver inflammation. Endotoxin can also directly stimulate HSCs and induce liver fibrosis via transforming growth factor-β signaling [19]. In the present study, hepatic expression of LBP, which directly correlates with portal endotoxin levels, and TLR4, the main downstream signal of LBP in the liver, were highest in the CDAA + fructose group. Also, our immunohistochemical examinations indicated that LPS stimulates not only Kupffer cells but HSCs. LPS is a cell-wall component of gram-negative bacteria which plays an important role in augmenting inflammation in NAFLD [20]. We previously reported that rats fed with a CDAA diet had lower levels of TJPs, which resulted in liver fibrosis by activation of LPS-TLR4 signaling [21]. In the current study, we have shown that oral fructose administration is associated with reduction of TJPs in the intestinal mucosa and with exacerbation of liver fibrosis. Fructose has been reported to reduce levels of serotonin reuptake transporter protein in the small intestine, a protein which is essential for regulating intestinal permeability [22]. Furthermore development of metabolic syndrome caused by fructose is directly correlated with variations of the gut content of specific bacterial taxa [12]. Interestingly, we have also examined the impact of oral administration of other major saccharides i.e. glucose or sucrose on CDAA diet-induced diseased liver, however, no significant histological alterations were observed by administrating these saccharides, suggesting the hepatic phenotypic observations shown this experiment is specific for fructose (data not shown). We have also demonstrated that oral administration of nonabsorbable antibiotics to CDAA-treated rats improved intestinal permeability and attenuated the development of liver fibrosis [21]. Taken together, our data indicate that fructose-induced leaky gut, perhaps in association with dysbiosis, plays an important role in the development of liver fibrosis by increasing the delivery of LPS to the liver.

Another concern with NASH is that it confers an increased risk of hepatocellular carcinoma (HCC) [23]. In the present study, the number of GST-P positive preneoplastic lesions was significantly higher in rats fed CDAA + fructose group in those fed CDAA without fructose, although the size of such lesions did not differ significantly between the two CDAA groups. Our findings suggest that fructose could augment hepatocarcinogenesis, although the exact mechanism remains unclear. One possibility is that the greater degree of liver fibrosis attributed to fructose administration contributes to an increased in HCC risk, as is seen in various chronic liver diseases [24]. Another possibility is that increased LPS-TLR4 signaling can augment hepatocarcinogenesis [25, 26]. Longer term experiments are needed to assess whether frank HCCs can be induced with fructose in this rat model.

Our previous study demonstrated that treatment with nonabsorbable antibiotics reduced the development of liver fibrosis in the rat CDAA model [21]. Taken together with our current findings, it is worth speculating whether control of the intestinal microbiota can reduce portal endotoxin levels, which could then result in reduced liver fibrogenesis and a lower risk of HCC. Supporting this hypothesis is the finding that TLR4-mutant mice were resistant to hepatic steatosis, induction of TNF-α, lipid peroxidation, and insulin resistance compared with wild type mice [20]. In clinical NASH, delivery of endotoxins from the intestinal tract through the portal vein is considered a potent factor by which fructose causes progression of NAFLD [10, 11]. These findings strongly support those of our current study, demonstrating that a fructose-induced leaky gut is a strong driving force for the progression of NASH by way of increased endotoxin-TLR4 signaling, which may result not only in increased fibrosis but also hepatocarcinogenesis.

The limitations of this study would be that this model lacks insulin resistance and obesity, both of which are major phenomena of clinical NASH. This reason could be explained the previous report that methionine- or choline-deficiency in mice augment severe progression of liver pathogenesis followed by a rapid formation of steatohepatitis due to strong inhibition of SCD-1, playing an important role in metabolism [27]. Further investigation using other rodent models of NASH is required to confirm whether these effects of fructose independently contribute to the exacerbation of liver fibrogenesis and hepatocarcinogenesis in NASH.

In conclusion, fructose administration in a rat model of NASH led to increased intestinal permeability because of attenuation of the expression of TJPs. This fructose-induced leaky gut was associated with a marked increase in liver fibrosis and the potential for hepatocarcinogenesis by induction of the LPS-TLR4 signaling cascade. Fructose thus can be considered as an important factor in multiple parallel hits hypothesis on the cause of progression of NASH.

## MATERIALS AND METHODS

### Animals and treatment

Male 6-week-old Fisher 344 rats (Japan SLC, Hamamatsu Japan) were housed in a room under controlled temperature and a 12/12 h light-dark cycle. Rats were divided into three groups based on free access for 10 weeks to one of three diets: 1) CSAA diet and plain tap water (CSAA group; n=4), 2) CDAA diet (CLEA Japan, Tokyo, Japan) and plain tap water (CDAA group; n=6), and 3) CDAA diet and water with 20% (w/v) fructose (CDAA + fructose group, n=6). The average of daily intake of food and water was assessed throughout the experimental period. Rats were killed at the end of 10 weeks. Body and liver weights were measured, and blood was collected from the portal vein. Specimens of the liver and small intestine were collected and immediately fixed in neutral-buffered formalin. This study was approved by the ethics committee of Nara Medical University, Kashihara, Japan.

### Histologic examination of liver specimens

Conventional histologic examination was performed with sirius-red (Narabyouri Research, Nara, Japan) staining of excised liver sections, as described previously [28]. Semi-quantitative analysis of sirius-red positive areas was performed using Image J software version 64 (National Institutes of Health, Bethesda, MD, USA).

### Immunohistochemistry

For immunohistostaining of αSMA and F4/80, 5 μm-thick liver sections were stained using the indirect immunoperoxidase technique with rabbit antimonoclonal antibody (Abcam, Cambridge, UK). For immunohistostaining of LBP, TLR4, pNF-κB, TNF-α and liver sections were stained likewise with antipolyclonal antibody (Abcam, Cambridge, UK). To calculate relative expression levels, 8 individual fields (original magnification ×200) of each tissue section were assessed.

GST-P, thought to be a marker of precancerous lesions of HCC [29], was stained using rabbit antimonoclonal anti-GST-P antibody (MBL, Nagoya, Japan). Three liver sections from each rat were placed on one slide and the average number and size of GST-P positive foci were measured.

Immunofluorescence-immunohistochemistry for ZO-1 was performed on paraffin-embedded intestinal sections using polyclonal antibodies to ZO-1 to assess TJPs (Invitrogen, Carlsbad, CA, USA). Immunofluorescence-immunohistochemistry for pNF-κB and αSMA was performed on paraffin-embedded liver sections using polyclonal antibodies as above. Detection of the primary antibodies was performed using 1:500 Alexa Fluor® conjugated secondary antibodies (Invitrogen).

### Real-time polymerase chain reaction (RT-PCR)

Total RNA was extracted from liver tissues using an RNeasy Mini Kit (Qiagen, Hilden, Germany) and transcribed to cDNA using a High Capacity RNA-to-cDNA Kit (Applied Biosystems, Foster City, CA, USA) according to the manufacturer’s manual. The hepatic mRNA levels of collagen1A1 (*Col1a1*), tumor growth factor beta 1 (*Tgfb1*), TLR4 (*Tlr4*), TNF-α (*Tnfa*), αSMA (*Acta2*), LBP (*Lbp*), fatty acid synthase (*Fas*), and sterol regulatory element-binding protein (*Srebp*) were measured using Applied Biosystems StepOnePlus Real-Time PCR (Applied Biosystems) with SYBR green (Applied Biosystems). Glyceraldehyde-3-phosphate dehydrogenase (*Gapdh*) expression was used as an endogenous reference for each sample. The cycling conditions were as follows: initial holding stage at 95°C for 20 sec, followed by 40 cycles at 95°C for 3 sec and 60°C for 30 sec, followed by the melting curve stage at 95°C for 15 sec, 60°C for 1 min, and 95°C for 15 sec.

Relative mRNA expression values were calculated using the relative standard curve method normalized to *Gapdh* expression. Each primer sequence was as follows: *Gapdh*, forward AGACAGCCGCATCTTCTTGT, reverse CTTGCCGTGGGTAGAGTCAT; *Tgfb1*, forward CGGCAGCTGTACATTGACTT, reverse AGCGCACGATCATGTTGGAC; *Col1a1*, forward TGCTGCCTTTTCTGTTCCTT, reverse AAGGTGCTGGGTAGGGAAGT; *Acta2* forward ACTGGGACGACATGGAAAAG, reverse CATCTCCAGAGTCCAGCACA; *Tlr4*, forward TGCTCAGACATGGCAGTTTC reverse TCAAGGCTTTTCCATCCAAC; *Lbp*, forward AAGGCGCAAGTGAGACTGAT, reverse AGTCGAGGTCGTGGAGCTTA; *Tnfa*, forward ACTCCCAGAAAAGCAAGCAA, reverse CGAGCAGGAATGAGAAGAGG; *Fas*, forward TCGAGACACATCGTTTGAGC, reverse TCAAAAAGTGCATCCAGCAG; *Srebp*, forward GTGGTCTTCCAGAGGCTGAG, reverse GGGTGAGAGCCTTGAGACAG.

### Determination of rat intestinal permeability

A total of 20 mg FITC-dextran (40kDa: Sigma-Aldrich, St.Louis, MO, USA) was orally administered to each rat 4 h before sacrifice. Blood was collected from the portal vein. To evaluate the degree of gut permeability, the plasma was analyzed by fluorescence measurement at an excitation wavelength of 490 nm and emission wavelength of 520 nm to detect the concentration of FITC-labeled dextran.

### Oxidative stress

Lipid peroxidation is an indicator of oxidative stress [30, 31]. Higher ROS concentrations are followed by hepatic fat accumulation, and hepatic lipid peroxidation plays a central role in the progression of NASH [32]. Hepatic TBARS, indicating the occurrence of lipid peroxidation, were measured using a TBARS measurement kit (Cayman Chemical, Ann Arbor, MI, USA) following the manufacturer’s manual.

### Statistical analysis

Data are expressed as the mean ± standard deviation. Variance between each experimental group was analyzed by an analysis of variance test. A P value <0.05 was considered to indicate a statistically significant difference.

## Abbreviations

NASH: Non-alcoholic steatohepatitis
CSAA: choline-supplemented/L-amino acid
CDAA: choline-deficient/L-amino acid
LPS: lipopolysaccharide
αSMA: alpha smooth muscle actin
TBARS: thiobarbituric acid reactive substances
TLR4: Toll-like receptor 4
LBP: LPS-binding protein
NF-κB: nuclear factor-kappa B
GST-P: glutathione S-transferase placental form
TJP: Tight junction protein
HSC: hepatic stellate cell
